# Impacts of climate-induced hydro-meteorological hazards on women’s reproductive and maternal health in India: An assessment of gender-based violence across stages of women's lives

**DOI:** 10.1016/j.joclim.2025.100630

**Published:** 2026-05-02

**Authors:** Saif Nihal, Anjali Sharma, Amit Mitra, Soumya Swaminathan, Nitya Rao

**Affiliations:** a Research Fellow, International Institute for Population Sciences (IIPS), Mumbai, India; b Independent Research Consultant, India; c Chairperson, M S Swaminathan Research Foundation (MSSRF), India; d Professor, School of Global Development, University of East Anglia, Norwich, NR4 7TJ, United Kingdom

**Keywords:** Climate change, Hydro-meteorological hazards, Gender-based violence, Geospatial analysis, India

## Abstract

**Introduction:**

Climate change, a global challenge with diverse manifestations, is often studied as a homogeneous phenomenon. Yet, different climate-change-induced hazards have differentiated implications for health system disruptions, social vulnerabilities, and reduced access to care. Understanding these links from a gendered perspective remains an urgent need. This study assesses the impacts of different hydro-meteorological hazards on gender-based violence (GBV) in India, identifying spatial hotspots where exposure converges with different forms of GBV. Our conceptualisation of GBV goes beyond traditional notions that focus on Intimate Partner Violence (IPV) to include the violation of rights in terms of early marriage or reduced access to reproductive health services.

**Materials and Methods:**

Data on exposure to extreme hydro-meteorological hazards and women’s wellbeing are obtained from India's Council on Energy, Environment, and Water (CEEW) and the fourth and fifth rounds of the National Family Health Survey (NFHS), respectively. Hotspots with high climate exposure and GBV were identified through geospatial analysis. The association between different hydro-meteorological hazards and GBV was determined through pooled logistic regression analysis.

**Results:**

Exposure to cyclones, a sudden-onset hazard, is associated with a higher likelihood of dropout from the minimum recommended Antenatal Care (60 %), while exposure to drought, a slow-onset hazard, is likely to increase dropouts by 38 % compared to non-exposure. The likely increase in intimate partner violence (IPV) is similar for drought (26 %) and cyclones (27 %). The impacts of floods are found to be lower on all indicators of GBV, with further in-depth studies of flood-affected areas needed to identify the causes. Districts in northern India are vulnerable to all types of GBV; however, some southern Indian districts are hotspots for girl child marriage and IPV.

**Discussion:**

This study shows that the association of hydro-meteorological hazards with GBV varies according to the type of hazard and its potential impacts on economic and livelihood disruptions, displacement, stress, and gender norms.

**Conclusion:**

This study indicates the need for hazard-specific targeted interventions from a gendered perspective, particularly in spatial hotspots.

## 1 Introduction

Climate change increases mortality, morbidity, malnutrition, and enhances susceptibility to various health conditions, with economic consequences for households and communities [1]. Considering the variability of intensity and impacts of sudden or slow onset climate events, adopting a heterogeneous, disaggregated analytical framework becomes crucial for a comprehensive assessment.

A review of 130 studies by the Global Gender and Climate Alliance suggests that women are disproportionately affected by natural disasters and climate stress due to higher poverty levels and dependence on local natural resources-based livelihoods [[2], [3], [4]]. Climate change is a “threat multiplier” [5], that intensifies pre-existing challenges in women’s lives through a complex interplay of socioeconomic, political and legal barriers, cultural norms and gender dynamics, that together shape women's vulnerabilities [[6], [7], [8], [9]]. Climate change has a higher negative impact on women-headed households and women-cultivated crops [10], with women disproportionately experiencing different forms of gender-based violence (GBV) [11].

GBV occurs throughout women’s lifecycle and impacts their overall health outcomes [12]. This manifests in the form of sex-selective abortions, female infanticide, girl child marriage, sexual abuse, domestic violence, differential access to care, nutrition, health and economic abuse [[13], [14], [15]]. Annually around 303,000 women die due to preventable causes associated with pregnancy and childbirth; 18 % of early adolescent girls experience sexual abuse; 12 million girls experience child marriage; women are more likely to be affected by infectious diseases and experience many health problems in their post-reproduction years [16]. The United Nations Population Fund (UNFPA) estimates that intimate partner violence (IPV) may increase three times in Sub-Saharan Africa due to climate change and rising temperatures by 2060 [17]. India has already seen a 6.23 % increase in IPV with each degree Celsius increase in annual temperature, higher than its South Asian neighbours [18].

In this paper, we analyse climate change impacts on key stages of women’s lives. We seek to (a) identify the spatial hotspots where high levels of climate change and GBV co-exist and (b) analyse the differential impacts of climate-induced hazards on women’s lives. The study makes at least two unique contributions: first, it analyses the impact of climate change on different dimensions of violence across women’s lifecycle, and second, it provides a disaggregated understanding of the impacts of different climate-induced hazards. No previous studies, to our knowledge, have analysed climate change and violence against women from this perspective.

## 2 Conceptual framework

**Fig. 1:**
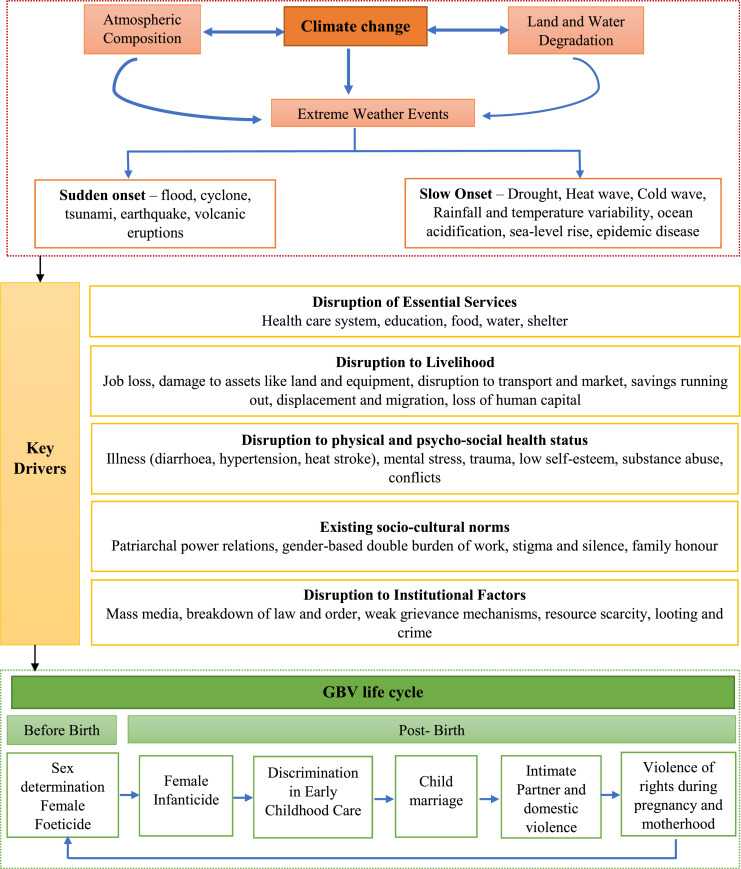
Conceptual Framework showing how climate change may exacerbate gender-based violence across women’s lifecycle.

Aligned with the World Bank, International Center for Research on Women (ICRW), UNFPA India and Organisation for Economic Co-operation and Development (OECD) frameworks, this study examines GBV across marriage, partner relationships, and motherhood. Fig. 1 presents the conceptual framework linking climate change to GBV, with impacts varying by the type of climate event - slow or sudden onset. Several drivers mediate the violence and health outcomes for women [19,20]. We consider three specific kinds of GBV: (1) child marriage, 2) IPV, and (3) violence of rights during pregnancy and motherhood.

Loss of assets, reduced opportunities for income generation, displacement of people, disruption to education and fear of sexual violence, exacerbated by extreme poverty and gender inequality, appear to be key factors leading to child marriages during an environmental crisis [21,22]. IPV is driven by climate-induced stressors including resource scarcity, loss of livelihood, displacement and food insecurity, alongside existing gender norms and inadequate support networks [23]. For motherhood, climate change may result in adverse pregnancy outcomes due to food and water insecurity, conflicts, displacement, poor health systems and a discriminatory socio-cultural environment. The pathways include stress, mental health problems, malnutrition and infections [24].

## 3 Materials and methods

### 3.1 Data source

We used two key data sources: (a) district-level data from the Council on Energy, Environment, and Water (CEEW) published in 2021 [25] for 640 districts, and (b) individual-level data from the fourth and fifth rounds of the National Family Health Survey (NFHS) conducted in 2015–16 and 2019–21. These datasets provide comprehensive insights into climate vulnerability at the district level and the well-being status of women within those districts [26].

### 3.2 Description of variables

We assessed women’s well-being with four major outcome variables: (1) girl child marriage, (2) IPV (3) miscarriage/stillbirth, and (4) drop-out from the minimum recommended Antenatal Care (ANC). Linked to women’s life-course, they reflect violence during marriage, partner relationships, and motherhood respectively.

The major predictor variable is climate vulnerability, which is assessed through two indicators: (1) exposure scores (indicating climate change) and (2) exposure to a particular hydro-meteorological hazard, namely, drought, flood, and/or cyclone. The first indicator identifies the spatial hotspots where climate change may exacerbate women’s vulnerabilities. The second indicator is used in bivariate and multivariate analysis to understand the heterogenous association of different hydro-meteorological hazards (droughts, floods, cyclone) with women’s wellbeing. The detailed definition of outcome and main predictor variables are given in Table A1.

The control variables, all drawn from the NFHS, include the place of residence, social group, wealth status, households with sanitation facility, clean water, clean fuel, mass media exposure, women's education, number of household members, and year of the survey.

### 3.3 Statistical analysis

The statistical analysis was conducted in two stages. First, geospatial analysis was undertaken using Geoda version 1.20 and Arc GIS to identify the hotspots where climate change (exposure scores) coexists with gender-based violence indicators. Local Indicators of Spatial Association (LISA) and the Moran’s I statistic are geospatial techniques utilised to analyse spatial autocorrelation among variables. Our focus was to identify the significant clusters rather than understanding overall spatial autocorrelation; therefore, LISA derived maps were used to visualise patterns of clustering, dispersion, or outliers in relation to space (in our case, districts) based on the Local Moran’s I statistics. The statistical significance of spatial autocorrelation is assessed through Monte Carlo randomisation approach with 999 permutations and presented at 95 % confidence intervals [27]. Second, a pooled logistic multivariate regression was carried out with separate models run for drought, floods and cyclones with each of the women's well-being indicators. Adjusted Odds Ratio (AOR) were estimated using STATA 16 software. Hotspots were identified for overall climate change, while the multivariate analysis was conducted to understand how the well-being of women surveyed from 2015 to 2021 is associated differently with different climate-change-induced hazards.

## 4 Results and discussions

### 4.1 Descriptive statistics

The mean exposure score of 0.40 implies that around 40 % of sampled women are exposed to climate change in India. Between 2010–19, around 60 % were exposed to drought, 36 % to flood and 26 % to cyclones. The prevalence of child marriage is 41 %, IPV 31 %, miscarriage/stillbirth 7 % and dropout from the minimum recommended ANC 34 %. Table 1

**Table 1:** Descriptive statistics of key independent and dependent variables.

Variable	Total Sample (N)	Mean	Standard Deviation	Minimum	Maximum
Exposure Score^a^	10,35,927	0.40	0.34	0	1
Exposure to drought	14,23,801	0.60	0.49	0	1
Exposure to flood	14,23,801	0.36	0.48	0	1
Exposure to Cyclone	14,23,801	0.26	0.44	0	1
Girl Child Marriage	10,42,445	0.41	0.49	0	1
IPV	1,29,864	0.31	0.46	0	1
Miscarriage/Still Birth	4,92,547	0.07	0.26	0	1
Drop out from minimum recommended ANC^b^	2,31,339	0.34	0.47	0	1

Note: ^a^Inter-quartile range of exposure score is 0.70, other variables are binary

b only for most recent birth

### 4.2 Spatial hotspots

**Fig. 2:**
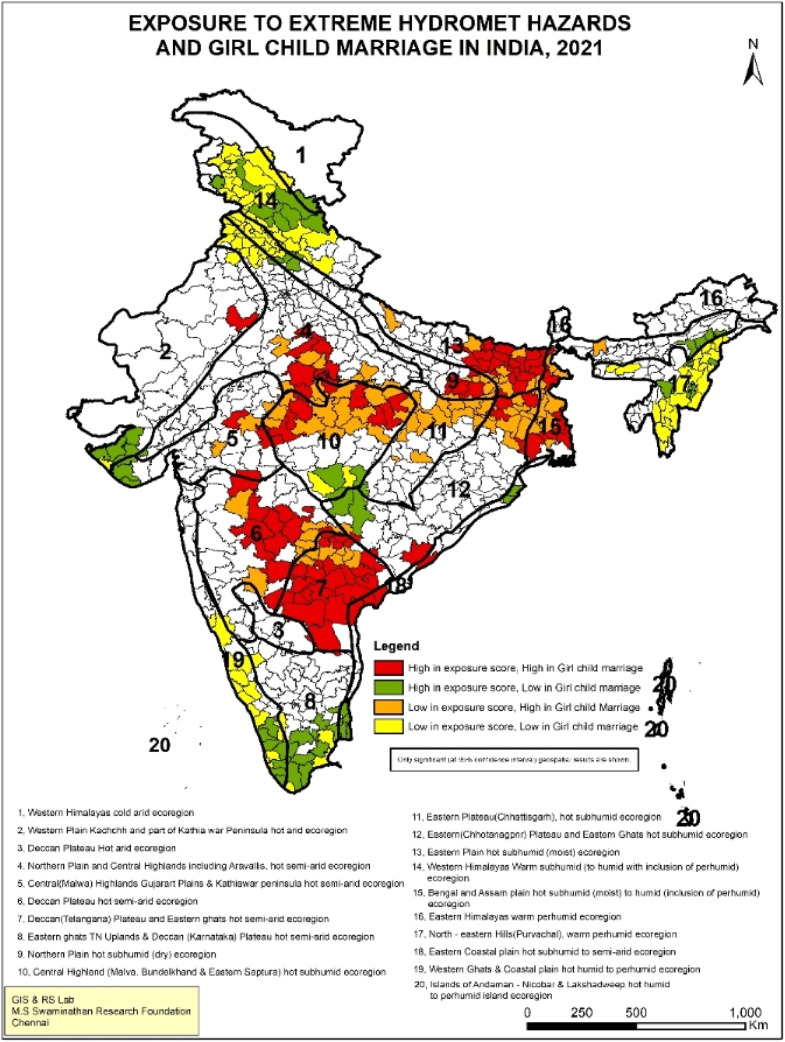
Geospatial analysis of exposure to extreme hydro-meteorological hazards and girl-child marriage.

The geospatial analysis reveals that most districts of Andhra Pradesh, Bihar, and parts of Maharashtra are major hotspots with a high prevalence of girl child marriage and high exposure scores for extreme hydro-meteorological hazards. Coexistence of high girl child marriage and high exposure scores also prevail in parts of southern West Bengal, Madhya Pradesh, Uttar Pradesh and Telangana (Fig. 2). Most studies on girl child marriage show a strong relationship with high poverty and low education levels [28,29], as reflected also in our analysis in Table 2, Table 3, Table 4, alongside patriarchal socio-cultural norms. While there are several studies for northern India, we found only two studies for southern India, both conducted in Andhra Pradesh and Telangana. One of these suggested that customary practices, including pressure from elders during specific festivals considered auspicious for marriage, is a major reason for early marriage of the girl child [30]. The second study points to the custom of daughters in the family being married before sons, even if the sons are older. Prospective deaths in the family also increase the chances of early marriage, as no auspicious occasion can be celebrated for several years following a death in the household [31]. Hotspots identified with girl child marriage in our study align with already vulnerable regions in India [32], implying that the intersection of areas with high girl child marriage and high exposure scores to hydro-meteorological hazards may exacerbate the existing vulnerabilities if not addressed.

**Table 2:** Adjusted Odds Ratio showing the association of women’s well-being with exposure to drought in India.

	Girl Child Marriage	Intimate Partner Violence	Miscarriage/ Stillbirth	Drop out from minimum recommended ANC
**Exposure to drought**				
No	Reference	Reference	Reference	Reference
Yes	1.03*** (1.02–1.05)	1.26*** (1.21–1.31)	1.06** (1.02–1.11)	1.38*** (1.34–1.43)
**Place of Residence**				
Urban	Reference	Reference	Reference	Reference
Rural	1.10*** (1.08–1.12)	0.90*** (0.85–0.94)	1.02 (0.98–1.07)	1.21*** (1.17–1.26)
**Social Group**				
Scheduled Caste (SC)	Reference	Reference	Reference	Reference
Scheduled Tribe (ST)	0.83*** (0.81–0.84)	0.79*** (0.74–0.83)	0.64*** (0.6–0.67)	0.76*** (0.72–0.8)
Other Backward Class (OBC)	0.94*** (0.93–0.96)	0.89*** (0.85–0.93)	0.97 (0.94–1.01)	1.04* (1.01–1.08)
Uncategorised	0.92*** (0.90–0.93)	0.70*** (0.66–0.75)	1.00 (0.95–1.05)	0.90*** (0.86–0.94)
**Wealth Status**				
Poorest	Reference	Reference	Reference	Reference
Poorer	0.97** (0.95–0.99)	0.83*** (0.79–0.87)	1.15*** (1.09–1.2)	0.75*** (0.72–0.78)
Middle	0.89*** (0.87–0.90)	0.68*** (0.64–0.72)	1.17*** (1.11–1.24)	0.62*** (0.59–0.64)
Richer	0.75*** (0.73–0.77)	0.54*** (0.5–0.58)	1.20*** (1.12–1.28)	0.57*** (0.54–0.6)
Richest	0.55*** (0.53–0.57)	0.35*** (0.32–0.38)	1.32*** (1.22–1.43)	0.52*** (0.49–0.56)
**Sanitation Facility**				
No improved facility	Reference	Reference	Reference	Reference
Improved Sanitation Facility	0.98 (0.97–1.00)	0.89*** (0.85–0.94)	1.04 (1–1.08)	0.91*** (0.88–0.94)
**Clean water facility**				
No improved facility	Reference	Reference	Reference	Reference
Improved Source of drinking water	0.93*** (0.91–0.95)	1.11** (1.04–1.18)	1.15*** (1.08–1.23)	1.12*** (1.06–1.18)
**Clean cooking Fuel**				
No improved facility	Reference	Reference	Reference	Reference
Improved Cooking Fuel	1.07*** (1.05–1.08)	1.18*** (1.12–1.23)	0.88*** (0.84–0.92)	0.87*** (0.84–0.9)
**Mass Media Exposure**				
No	Reference	Reference	Reference	Reference
Yes	0.95*** (0.93–0.96)	1.07** (1.02–1.12)	1.03 (0.99–1.07)	0.58*** (0.57–0.6)
**Women's Education**				
<10 years of schooling	Reference	Reference	Reference	Reference
10+ years of schooling	0.29*** (0.29–0.30)	0.65*** (0.62–0.68)	0.92*** (0.89–0.96)	0.78*** (0.76–0.81)
**Number of household members**				
<5 members	Reference	Reference	Reference	Reference
5 or >5 members	1.09*** (1.07–1.10)	0.96* (0.92–0.99)	0.79*** (0.76–0.82)	1.24*** (1.2–1.28)
**Time**	0.92*** (0.91–0.93)	0.91*** (0.88–0.95)	1.15*** (1.11–1.19)	0.97 (0.94–1)
Intercept (constant)	1.39*** (1.34–1.44)	1.22* (1.01–1.48)	0.04*** (0.03–0.05)	0.83* (0.71–0.96)
N	9,57,615	1,20,389	4,44,632	2,07,286

Note: ***<0.001, **<0.01, *<0.05; CI at 95 % in parentheses (); Reference point is 1.00.

**Table 3:** Adjusted Odds Ratio showing the association of women’s well-being with exposure to floods in India.

	Girl Child Marriage	Intimate Partner Violence	Miscarriage/ Stillbirth	Drop out from minimum recommended ANC
**Exposure to flood**				
No	Reference	Reference	Reference	Reference
Yes	1.00 (0.99–1.02)	1.07*** (1.04–1.11)	1.02 (0.99–1.05)	1.07*** (1.04–1.1)
**Place of Residence**				
Urban	Reference	Reference	Reference	Reference
Rural	1.10*** (1.08–1.12)	0.91*** (0.86–0.95)	1.03 (0.98–1.07)	1.23*** (1.19–1.28)
**Social Group**				
Scheduled Caste (SC)	Reference	Reference	Reference	Reference
Scheduled Tribe (ST)	0.83*** (0.81–0.85)	0.79*** (0.74–0.84)	0.64*** (0.6–0.67)	0.76*** (0.73–0.8)
Other Backward Class (OBC)	0.95*** (0.93–0.96)	0.91*** (0.87–0.95)	0.98 (0.94–1.02)	1.07*** (1.04–1.11)
Uncategorised	0.91*** (0.89–0.93)	0.68*** (0.64–0.73)	0.99 (0.94–1.05)	0.87*** (0.83–0.91)
**Wealth Status**				
Poorest	Reference	Reference	Reference	Reference
Poorer	0.97** (0.95–0.99)	0.84*** (0.79–0.88)	1.15*** (1.1–1.21)	0.76*** (0.73–0.79)
Middle	0.89*** (0.87–0.91)	0.7*** (0.66–0.74)	1.18*** (1.12–1.25)	0.64*** (0.61–0.67)
Richer	0.75*** (0.74–0.77)	0.56*** (0.52–0.6)	1.21*** (1.13–1.3)	0.6*** (0.57–0.64)
Richest	0.55*** (0.53–0.57)	0.36*** (0.33–0.4)	1.33*** (1.23–1.44)	0.55*** (0.51–0.59)
**Sanitation Facility**				
No improved facility	Reference	Reference	Reference	Reference
Improved Sanitation Facility	0.98* (0.96–1)	0.87*** (0.83–0.91)	1.03 (0.99–1.07)	0.87*** (0.85–0.9)
**Clean water facility**				
No improved facility	Reference	Reference	Reference	Reference
Improved Source of drinking water	0.93*** (0.91–0.95)	1.11** (1.04–1.18)	1.15*** (1.08–1.23)	1.11*** (1.06–1.17)
**Clean cooking Fuel**				
No improved facility	Reference	Reference	Reference	Reference
Improved Cooking Fuel	1.07*** (1.05–1.09)	1.18*** (1.13–1.24)	0.88*** (0.84–0.92)	0.87*** (0.84–0.9)
**Mass Media Exposure**				
No	Reference	Reference	Reference	Reference
Yes	0.95*** (0.93–0.96)	1.07** (1.02–1.12)	1.03 (0.99–1.07)	0.59*** (0.57–0.61)
**Women's Education**				
<10 years of schooling	Reference	Reference	Reference	Reference
10+ years of schooling	0.29*** (0.29–0.3)	0.65*** (0.62–0.68)	0.92*** (0.89–0.96)	0.79*** (0.76–0.81)
**Number of household members**				
<5 members	Reference	Reference	Reference	Reference
5 or >5 members	1.09*** (1.08–1.1)	0.96* (0.93–0.99)	0.79*** (0.76–0.82)	1.24*** (1.21–1.28)
**Time**	0.92*** (0.91–0.93)	0.91*** (0.88–0.95)	1.15*** (1.11–1.19)	0.97* (0.94–1)
Intercept (constant)	1.99*** (1.86–2.13)	1.38** (1.14–1.66)	0.04*** (0.04–0.05)	1.02 (0.88–1.18)
N	9,57,615	1,20,389	4,44,632	2,07,286

Note: ***<0.001, **<0.01, *<0.05; CI at 95 % in parentheses (); Reference point is 1.00.

**Table 4:** Adjusted Odds Ratio showing the association of women’s well-being with exposure to cyclones in India.

	Girl Child Marriage	Intimate Partner Violence	Miscarriage/ Stillbirth	Drop out from minimum recommended ANC
**Exposure to cyclones**				
No	Reference	Reference	Reference	Reference
Yes	1.10*** (1.09–1.12)	1.27*** (1.22–1.32)	1.26*** (1.22–1.3)	1.60*** (1.56–1.65)
**Place of Residence**				
Urban	Reference	Reference	Reference	Reference
Rural	1.10*** (1.08–1.12)	0.91*** (0.87–0.96)	1.03 (0.98–1.08)	1.24*** (1.19–1.29)
**Social Group**				
Scheduled Caste (SC)	Reference	Reference	Reference	Reference
Scheduled Tribe (ST)	0.84*** (0.82–0.86)	0.81*** (0.76–0.86)	0.66*** (0.62–0.7)	0.80*** (0.76–0.84)
Other Backward Class (OBC)	0.94*** (0.93–0.96)	0.9*** (0.86–0.94)	0.96 (0.93–1)	1.05* (1.01–1.08)
Uncategorised	0.91*** (0.89–0.93)	0.68*** (0.64–0.72)	0.99 (0.94–1.04)	0.87*** (0.83–0.91)
**Wealth Status**				
Poorest	Reference	Reference	Reference	Reference
Poorer	0.98* (0.96–1.00)	0.86*** (0.81–0.90)	1.18*** (1.13–1.24)	0.79*** (0.76–0.83)
Middle	0.9*** (0.88–0.92)	0.72*** (0.68–0.77)	1.24*** (1.17–1.31)	0.69*** (0.66–0.72)
Richer	0.77*** (0.75–0.79)	0.58*** (0.54–0.63)	1.28*** (1.19–1.37)	0.66*** (0.62–0.7)
Richest	0.56*** (0.55–0.58)	0.38*** (0.35–0.42)	1.42*** (1.31–1.54)	0.61*** (0.57–0.65)
**Sanitation Facility**				
No improved facility	Reference	Reference	Reference	Reference
Improved Sanitation Facility	0.98* (0.96–0.99)	0.87*** (0.83–0.91)	1.03 (0.99–1.07)	0.87*** (0.84–0.9)
**Clean water facility**				
No improved facility	Reference	Reference	Reference	Reference
Improved Source of drinking water	0.93*** (0.91–0.95)	1.09** (1.03–1.16)	1.12* (1.05–1.19)	1.07** (1.02–1.13)
**Clean cooking Fuel**				
No improved facility	Reference	Reference	Reference	Reference
Improved Cooking Fuel	1.06*** (1.05–1.08)	1.18*** (1.13–1.24)	0.87*** (0.84–0.91)	0.86*** (0.83–0.89)
**Mass Media Exposure**				
No	Reference	Reference	Reference	Reference
Yes	0.95*** (0.94–0.97)	1.09*** (1.04–1.14)	1.06* (1.02–1.1)	0.61*** (0.59–0.63)
**Women's Education**				
<10 years of schooling	Reference	Reference	Reference	Reference
10+ years of schooling	0.29*** (0.29–0.3)	0.65*** (0.62–0.68)	0.92*** (0.88–0.95)	0.77*** (0.75–0.8)
**Number of household members**				
<5 members	Reference	Reference	Reference	Reference
5 or >5 members	1.09*** (1.07–1.1)	0.96* (0.92–0.99)	0.79*** (0.76–0.82)	1.25*** (1.21–1.29)
**Time**	0.92*** (0.91–0.93)	0.91*** (0.88–0.95)	1.16*** (1.12–1.19)	0.97* (0.94–1)
Intercept (constant)	1.89*** (1.77–2.02)	1.27* (1.05–1.54)	0.04*** (0.03–0.04)	0.85* (0.73–0.99)
N	9,57,615	1,20,389	4,44,632	2,07,286

Note: ***<0.001, **<0.01, *<0.05; CI at 95 % in parentheses (); Reference point is 1.00.

**Fig. 3:**
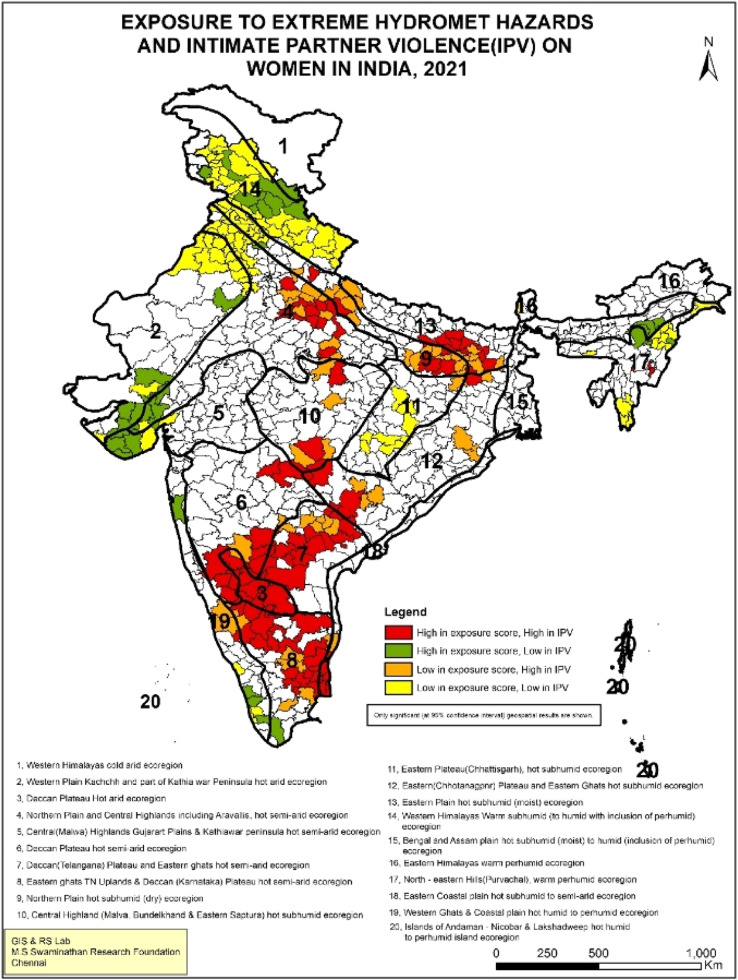
Geospatial analysis of exposure to extreme hydro-meteorological hazards and prevalence of IPV.

**Fig. 4:**
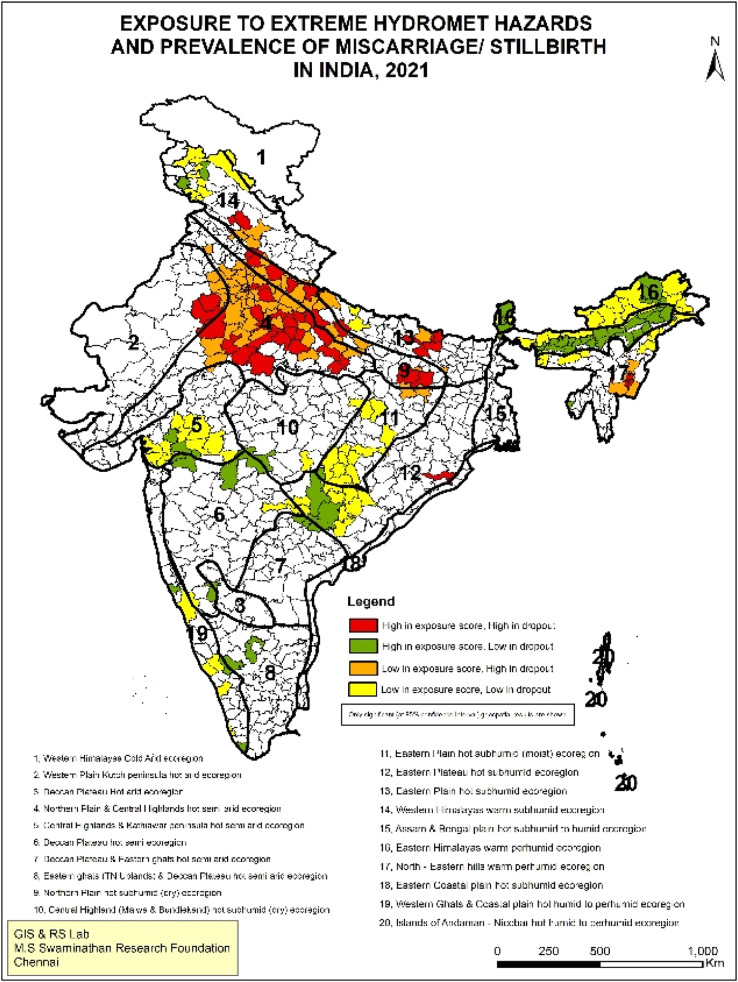
Geospatial analysis of exposure to extreme hydro-meteorological hazards and prevalence of miscarriage/stillbirth.

**Fig. 5:**
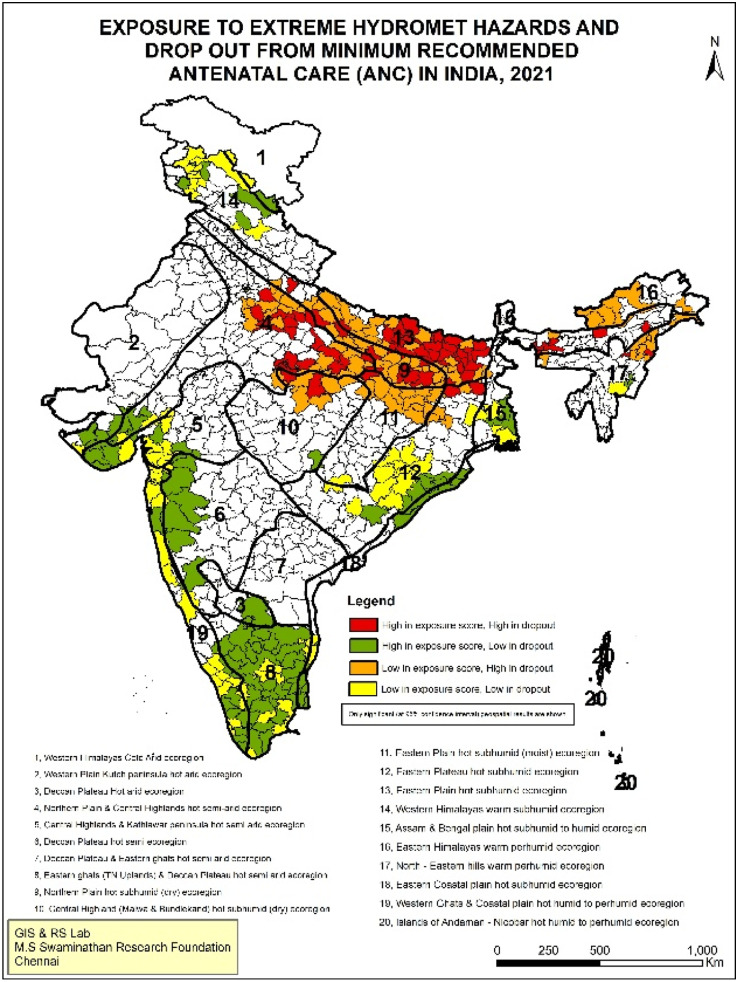
Geospatial analysis of exposure to extreme hydro-meteorological hazards and drop out from minimum recommended ANC.

This is also evident for IPV. The spatial overlap of high IPV and high exposure scores to extreme hydro-meteorological hazards are particularly clustered in southern India, including Karnataka, Andhra Pradesh, Telangana, Tamil Nadu and parts of Maharashtra. In northern India, parts of Bihar and Uttar Pradesh witness coexistence (Fig. 3). These hotspots align with regions in India most vulnerable to IPV, spread across central, eastern, and southern India [33]. While attributed primarily to multi-dimensional poverty, southern India remains a paradox of high IPV with low poverty as well as high levels of women’s education [34]. Likewise, the affected areas for maternal health indicators, i.e. miscarriage/stillbirth and dropout from the minimum recommended ANC visits with high exposure scores, lie in Uttar Pradesh and Bihar (Figs. 4& 5). Previous studies have also identified poor maternal health care in these states due to weak healthcare infrastructure, low human resources and low women’s education[35,36].

Overall, the geospatial analysis shows that the ‘high-high’ score (dark red-coloured patches) points to the co-existence of high exposure to climate change with poor women's well-being indicators. These hotspots require immediate action to mitigate the impacts of climate change. A ‘high-low’ score (green-coloured patches) indicates that while high exposure to climate change prevails, women's well-being indicators are comparatively better; these are the districts from which lessons can be learnt on addressing the impacts of climate change. Lastly, the ‘low-high’ score (orange-coloured patches) implies lower climate change exposure but a higher burden of women’s vulnerability. These are potential areas where climate change may have an impact in the future, so these regions should be made climate resilient. The significance map at 95 % confidence interval and scatterplot of LISA results are given in Supplemental File (Fig S.1-S.4.)

### 4.3 Association of extreme hydro-meteorological hazards with GBV

**Fig. 6:**
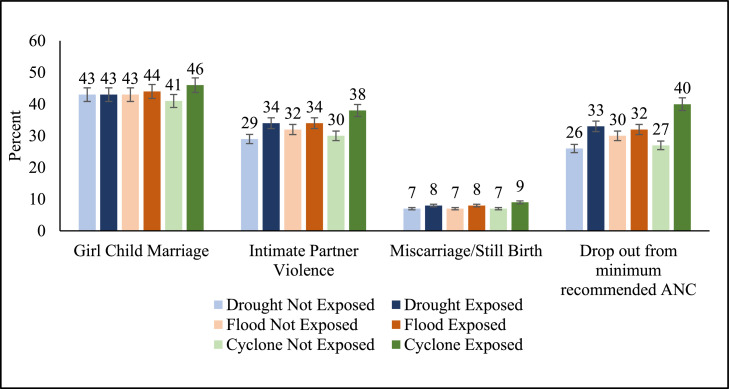
Percent distribution of women's well-being by exposure to hydro-meteorological hazards.

Both bivariate and multivariate analyses reveal a significant association between extreme hydro-meteorological hazards and GBV, but the extent of association differs by the type of hazard. Fig. 6 shows that compared to floods and droughts, exposure to cyclones led to a higher level of drop-out from the minimum recommended ANC, increased prevalence of IPV and marginally higher vulnerability to girl child marriage and miscarriage/stillbirth. For example, the prevalence of drop-out from minimum recommended ANC for those exposed to drought is 33 %, compared to those exposed to cyclones at 40 %. Similarly, the prevalence of girl child marriage is 43 % for those exposed to drought, but 44 % and 46 % for those exposed to flood and cyclones respectively.

#### 4.3.1 Slow-onset extreme weather event – drought

Drought, a slow onset extreme weather event, perpetuates GBV through an interplay of women’s increased burden of work, resource scarcity and economic loss due to crop failure. Drought-exposed districts have a significantly higher likelihood of girl-child marriage compared to districts not exposed to drought (Table 2). A study in the drought-prone region of Marathwada reaffirmed that loss of livelihood due to insufficient rainfall forced people to migrate, driving early marriage in the region [37]. In Sub-Saharan Africa too, drought-related financial strain and resource scarcity are linked to early marriages [38,39]. Apart from financial pathways, added household responsibilities during droughts, like fetching water and longer working hours, force girls to drop out of school [[40], [41], [42], [43]] and may exacerbate child marriages. As reflected in Table 2, pre-existing vulnerabilities such as low education levels can lead to a higher level of girl child marriages.

Similarly, droughts may increase IPV. We found that the prevalence of IPV is 26 % more likely if exposed to droughts (Table 2), particularly among financially dependent women [44]. Additionally, we found that drought has a significant impact on women’s health outcomes and access to health services. The prevalence of miscarriage/stillbirth is 6 % more likely among those exposed to drought compared to those who are not. One possible reason for the increased risk of maternal complications, including miscarriage and stillbirth, could be that during adverse climatic conditions like droughts, women’s workloads increase including in procuring fuel, fodder, and water [45], accompanied by dietary imbalances [46]. Drought-induced water scarcity and extreme heat can lead to heat exhaustion, hypertension, dehydration, insomnia, and irritability, resulting in miscarriages and preterm births [47]. Surprisingly our analysis reveals that having improved water facility as shown in Table 2 may lead to increase in IPV, miscarriage/still birth and drop-out from minimum recommended ANC; this may be because the availability of water infrastructure does not ensure clean water due to dry taps during droughts.

Limited access to quality healthcare services in drought-affected areas further exacerbates the risk of adverse pregnancy outcomes [48]. We find a 38 % higher likelihood of drop-out from minimum recommended ANC in drought affected populations. This implies that drought-related challenges such as financial constraints, food scarcity and prioritisation of immediate needs over healthcare, together adversely affect women’s wellbeing. This is corroborated by Table 2 which shows that women belonging to the poorest households are likely to experience higher levels of GBV, with the exception of miscarriage/stillbirth.

While our analysis doesn’t disaggregate the impacts of these hydro-meteorological hazards by socio-economic group, given that drought intensifies pre-existing vulnerabilities, we may infer that across most of these indicators, those residing in rural areas, in particular, the Scheduled Castes, are likely to be worst affected.

#### 4.3.2 Sudden-onset extreme weather event – floods and cyclones

Floods and cyclones have immediate effects on lives, assets and infrastructure, and may increase the vulnerability of women. We find exposure to cyclones significantly increases the prevalence of girl-child marriage by 10 % (Table 4). Our findings on cyclones align with similar studies in Bangladesh, which highlight that poverty, dowry-related concerns and sociocultural norms are key drivers of child marriage in response to cyclones [49], along with fear of sexual violence in temporary shelters [50]. Apart from this, girl child marriage is found to be a strong coping mechanism for lowering household expenses [50,51]. While studies in Bangladesh also find a significant positive association between girl child marriage and floods [50,51], we do not find this association (Table 3). A study of the Kosi floods in India, however, notes that the impact of floods on the marriage market depends upon the extent of economic shock and property destruction, including of school infrastructure [52]. Table 3, Table 4 also point out that girl child marriage is likely to be higher among the poorest. Here again, rural women, in particular, those belonging to the Scheduled Castes, are likely to be worst affected, with cyclones and floods intensifying their pre-existing vulnerabilities.

We find that IPV is likely to significantly increase by 7 % if the population is exposed to floods and 27 % if exposed to cyclones compared to those not exposed (Table 3 & 4). Rai et al. (2021) confirm that women residing in districts affected by cyclones in India face higher odds of experiencing emotional, physical, and sexual violence compared to those unexposed [53]. Possible reasons include increased jealousy among male partners and fear of losing status in the family, due to economic loss, dissatisfaction with intimate relations in temporary shelters and the financial dependency of women [54,55].

Additionally, miscarriage/stillbirth is likely to increase by 26 % and dropout from minimum recommended ANC by 60 % for those exposed to cyclones, compared to those not exposed (Table 4), a finding that aligns with similar studies. In regions affected by El-Nino, for example, the risk of perinatal infant mortality increased due to the already weak health system and limited resilience [56]. Also, during disasters, the delivery of prenatal care becomes challenging due to infrastructural damage, for a health system already overburdened, risking pregnancy complications and unsafe childbirth, increasing maternal and infant morbidity and mortality [57], [58].

During floods, dropout from the minimum recommended ANC is likely to increase by 7 % compared to those unexposed, however, the association of miscarriage/stillbirth and floods is not significant (Table 3). The findings on ANC utilisation are also evident in other studies [58,59]. However, findings on miscarriage/stillbirths need further research as previous studies highlight that the limited availability of transport, damaged roads, disruption to supply chains for medicines, and shortage of female medical staff during floods significantly affect the health outcomes of women [60,61]. Vaginal infections during floods are another important element affecting women’s reproductive health [61].

## 5 Strengths and limitations

The empirical evidence provided by our study highlights the urgent need for gender-responsive and climate-resilient strategies to safeguard women's health and safety in disaster-prone regions, particularly in the identified spatial hotspots. Nevertheless, there are limitations in our research. First, this study focuses on hazards like droughts, floods, and cyclones but doesn’t consider the impacts of heat waves, air pollution, and variations in rainfall patterns on GBV in one frame due to a lack of nationally comparable data. Definitional changes over time of droughts and other hazards are also out of scope for our study. Second, the NFHS’s well-being data may not be collected immediately after extreme climatic events, making it difficult to capture the precise impact of hazards such as floods and cyclones. Also, hazards like droughts, floods, and cyclones often exhibit pronounced seasonal variability, with distinct patterns of occurrence and intensity across seasons. The study does not account for these seasonal variations in hazard exposure, potentially overlooking important temporal dynamics in climate vulnerability. Finally, migration is assumed to be a constant in this analysis.

## 6 Conclusion

This study examines the heterogeneous impacts of different climate-change-induced extreme hazards on GBV in India. Women’s well-being status is poor in certain regions of India. Its coexistence with high exposure scores to extreme hydro-meteorological hazards implies that climate change may intensify these pre-existing vulnerabilities. Further, findings reveal that droughts, cyclones, and floods do not have uniform consequences for women’s health and well-being; rather, each hazard manifests in distinct ways across different life stages. These impacts are also likely to vary by location and caste, with rural Scheduled Caste women being the worst affected across hazards and most indicators of violence, given their socio-economic marginalisation.

Cyclones are associated with higher disruptions in antenatal care and adverse pregnancy outcomes, as well as increased risks of IPV. Our study found droughts contribute to significant gaps in maternal healthcare service access, intensifying pre-existing inequalities in reproductive health, followed by an increased risk of IPV and child marriage. While floods have relatively lower associations with IPV, they still pose risks to women’s wellbeing.

These variations highlight that a uniform policy response to climate-induced hazards is inadequate. A hazard-specific approach is necessary to address differential vulnerabilities posed by each extreme event. Policies aimed at mitigating the effects of cyclones should prioritise ensuring continuity of reproductive healthcare during and after cyclone events followed by strengthening preventive and protective measures against GBV. Strategies for drought-prone regions must focus on building strong healthcare systems and developing long-term resilience in affected communities, as well as improving socio-economic conditions to overcome the slow and lesser visible impacts of droughts, such as girl child marriage. Flood-affected areas, though exhibiting relatively lower disruptions, require further studies to understand the root causes of GBV. The intersection of climate change and existing socio-economic disadvantages underscores the urgency of incorporating a gender-sensitive, hazard-specific perspective in policy formulation. By recognising the differential impacts of extreme events and focusing on spatial hotspots, targeted interventions can be developed that effectively reduce vulnerabilities and build resilience among the most at-risk populations.

## Data statement

The study is based on data from the National Family Health Survey (NFHS) (2015-16 and 2019-21), which are publicly available through the Demographic and Health Surveys (DHS) Program website (www.dhsprogram.com). The NFHS data are de-identified and do not contain any personally identifiable information about survey participants. Additionally, climate exposure data were obtained from the Council on Energy, Environment, and Water (CEEW) with permission directly from the organisation. All data used in this study adhere to ethical guidelines, and no personally identifiable information has been accessed or reported.

## Author agreement

The author have all worked together on this paper and agree to the terms and conditions of the journal, if the paper is accepted for publication.

## Funding

This research was supported by the Bill & Melinda Gates Foundation (BMGF). The funding agency had no role in the study design, data collection, analysis, interpretation, or manuscript preparation. The views expressed in this study are solely those of the authors.

## CRediT authorship contribution statement

**Saif Nihal:** Writing – original draft, Visualization, Validation, Software, Project administration, Investigation, Formal analysis, Data curation. **Anjali Sharma:** Writing – original draft, Visualization, Validation, Methodology, Investigation, Data curation. **Amit Mitra:** Writing – review & editing, Methodology, Conceptualization. **Soumya Swaminathan:** Writing – review & editing, Supervision, Resources, Project administration, Funding acquisition, Conceptualization. **Nitya Rao:** Writing – review & editing, Conceptualization.

## Declaration of competing interest

The authors declare that they have no known competing financial interests or personal relationships that could have appeared to influence the work reported in this paper.

## Figures and Tables

**Table A.1:** Description of the list of major predictors and outcome indicators.

Indicators	Definition	Data Source
Exposure Score	Exposure scores are developed using district-level profiling of India’s extreme climate events, including cyclones, floods, and droughts and their associated events through a pentad decadal spatio-temporal analysis of change in frequency and intensity. A higher score represents more vulnerability to climate change in response to higher changes in the frequency and intensity of hydro-meteorological hazards in that district. The United Nations International Strategy for Disaster Reduction (UNISDR) defines hydro-meteorological hazards as “the process or phenomenon of atmospheric, hydrological, or oceanographic nature that may cause loss of life, injury or other health impacts, property damage, loss of livelihoods and services, social and economic disruption, or environmental damage”. Only the hazards considered to fulfil at least one of the following criteria are considered: i) 10 or more people reported killed; ii) 100 or more people reported affected; iii) declaration of a state of emergency; iv) call for international assistance. The data for district-level profiling is taken from the Indian Meteorological Department (IMD), EM-DAT, World Meteorological Organisation (WMO) and PIB reports. Outliers are removed.	CEEW, 2021 based on 1970–2019 records
Exposure to drought	Droughts are defined as “an extended period of unusually low precipitation that produces a shortage of water, and operationally, it is defined as the degree of precipitation reduction that constitutes a drought, that varies by locality, climate and environmental sector” (EMDAT 2015). Drought is a recurring event. Droughts in India are categorised into three subtypes: i) meteorological drought, ii) hydrological drought, and iii) agricultural drought. Drought, described as a slow-onset process, is usually not associated with any other major climatic events apart from famines and heat waves. In this study, drought is defined in terms of its meteorological definition, that is, the deviation from normal rainfall for an extended period in a district.	CEEW based on records of 2010–19
Exposure to flood	Floods are defined as “a general term for the overflow of water from a stream channel onto normally dry land in the floodplain (riverine flooding), higher-than-normal levels along the coast and in lakes or reservoirs (coastal flooding) as well as ponding of water at or near the point where the rain fell (flash floods)” (EM-DAT 2015). The associated events considered in this study are i) landslides, ii) extreme rainfall, iii) hailstorms, iv) thunderstorms, and v) cloud bursts.	CEEW based on records of 2010–19
Exposure to cyclone	A cyclone is defined as a tropical storm originating over tropical or subtropical waters. Cyclones are characterised by a warm-core, non-frontal disturbance with a low-pressure centre, spiral rain bands, and strong winds. A cyclonic storm formed due to a monsoon depression may lead to extreme rainfall/hailstorms/floods/thunderstorms.	CEEW based on records of 2010–19
Girl Child Marriage	Percentage of ever-married women aged 15–49 years who got married before attaining the age of 18 years.	NFHS, 2015–16 and 2019–21
Intimate Partner Violence (IPV)	Percentage of ever-married women aged 18–49 years who experienced any kind of violence committed by their current and former husbands. Physical Violence involves pushing, shaking, or throwing something at her; slapping, twisting her arm or pulling her hair; punching her; kicking her, dragging her, or beating her up; trying to choke her or burn her on purpose; or threaten or attack her with any weapon. Sexual violence includes physically forcing her to have sexual intercourse even when she does not want to. Emotional violence includes saying or doing something to humiliate her in front of others, threatening to hurt or harm her or someone close to her; insulting her or making her feel bad about herself.	NFHS, 2015–16 and 2019–21
Miscarriage/ Still Birth	Percentage of women aged 15–49 years whose pregnancies ended in a non-live birth (miscarriage or stillbirth) in the five years preceding the survey	NFHS, 2015–16 and 2019–21
Dropout from minimum recommended antenatal care	Percentage of women aged 15–49 years who received timely initiation of antenatal care (ANC), i.e. within the first trimester (0–3 months), but didn’t receive sufficient recommended ANC visits i.e. at least four visits, for her most recent live birth in the five years preceding the survey.	NFHS, 2015–16 and 2019–21

## Appendix B Supplementary materials

Image, application 1
