# Supplementary material for: Impacts of climate-induced hydro-meteorological hazards on women’s reproductive and maternal health in India: An assessment of gender-based violence across stages of women's lives
Source: J Clim Chang Health. 2026 May 2;28:100630. doi: 10.1016/j.joclim.2025.100630 (PMC13147378; doi:10.1016/j.joclim.2025.100630)
Supplement: Supplementary file 1 [file mmc1.docx]

**Impacts of Climate-Induced Hydro-meteorological Hazards on Women’s Reproductive and Maternal Health in India: An assessment of gender-based violence across stages of women's lives**

**Supplemental File**

Figure S.1. LISA Scatter Plot matrix and Significance Map for Girl child Marriage


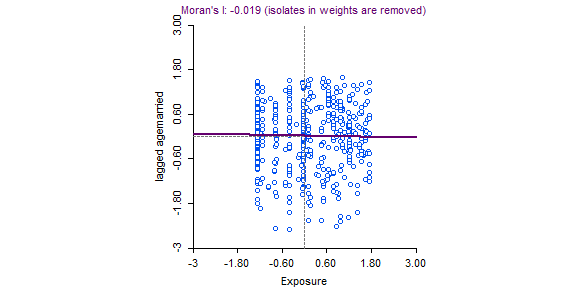

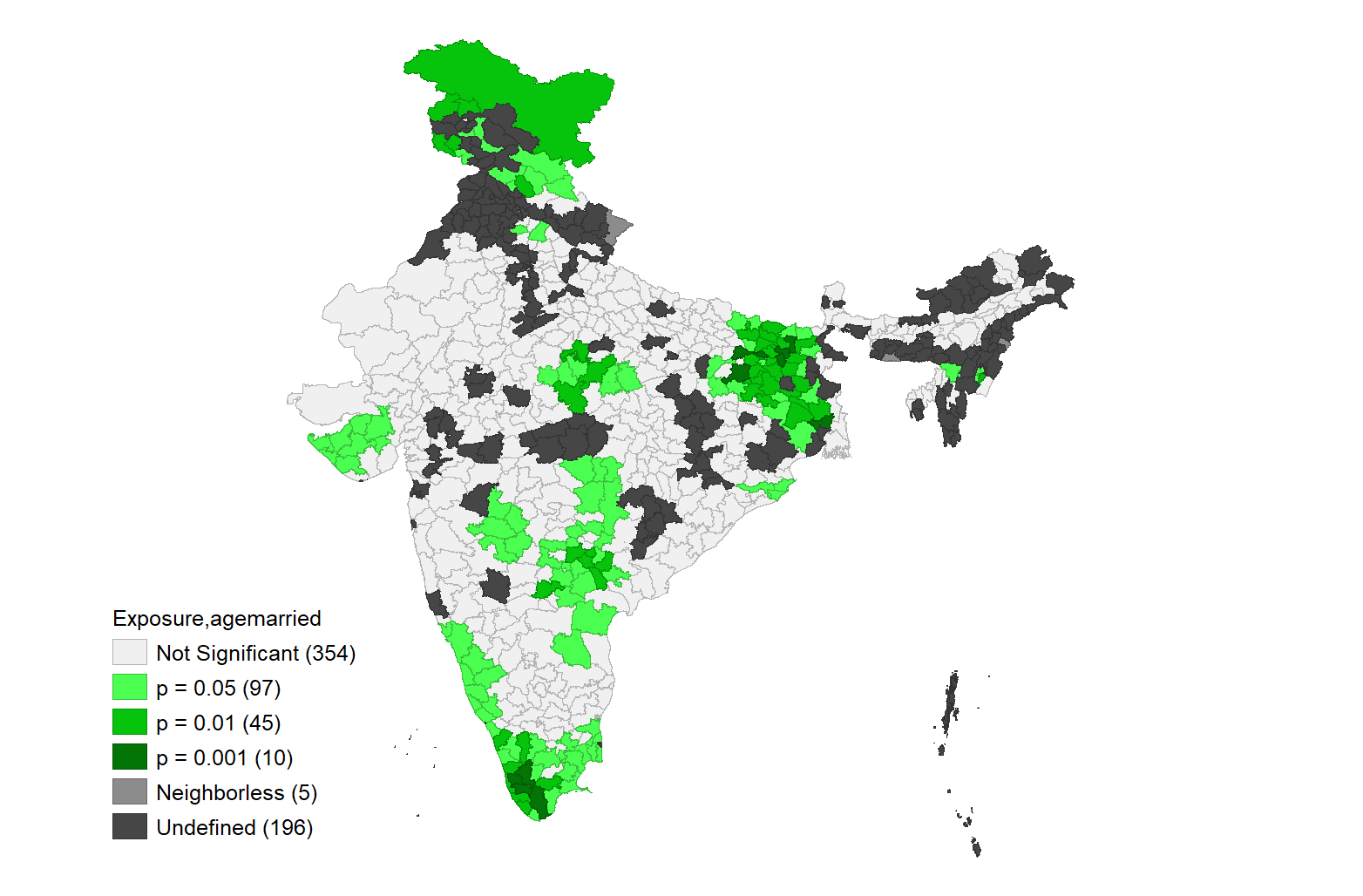


*Note: p-value for Global Moran’s I is 0.217*

Figure S.2. LISA Scatter Plot matrix and Significance Map for Intimate Partner Violence


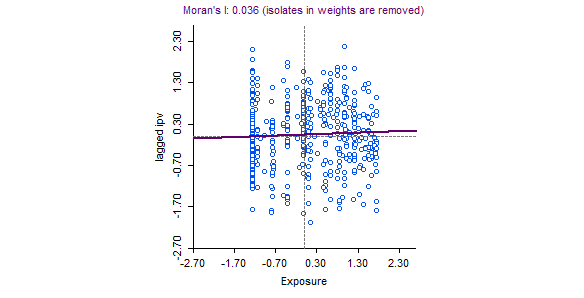

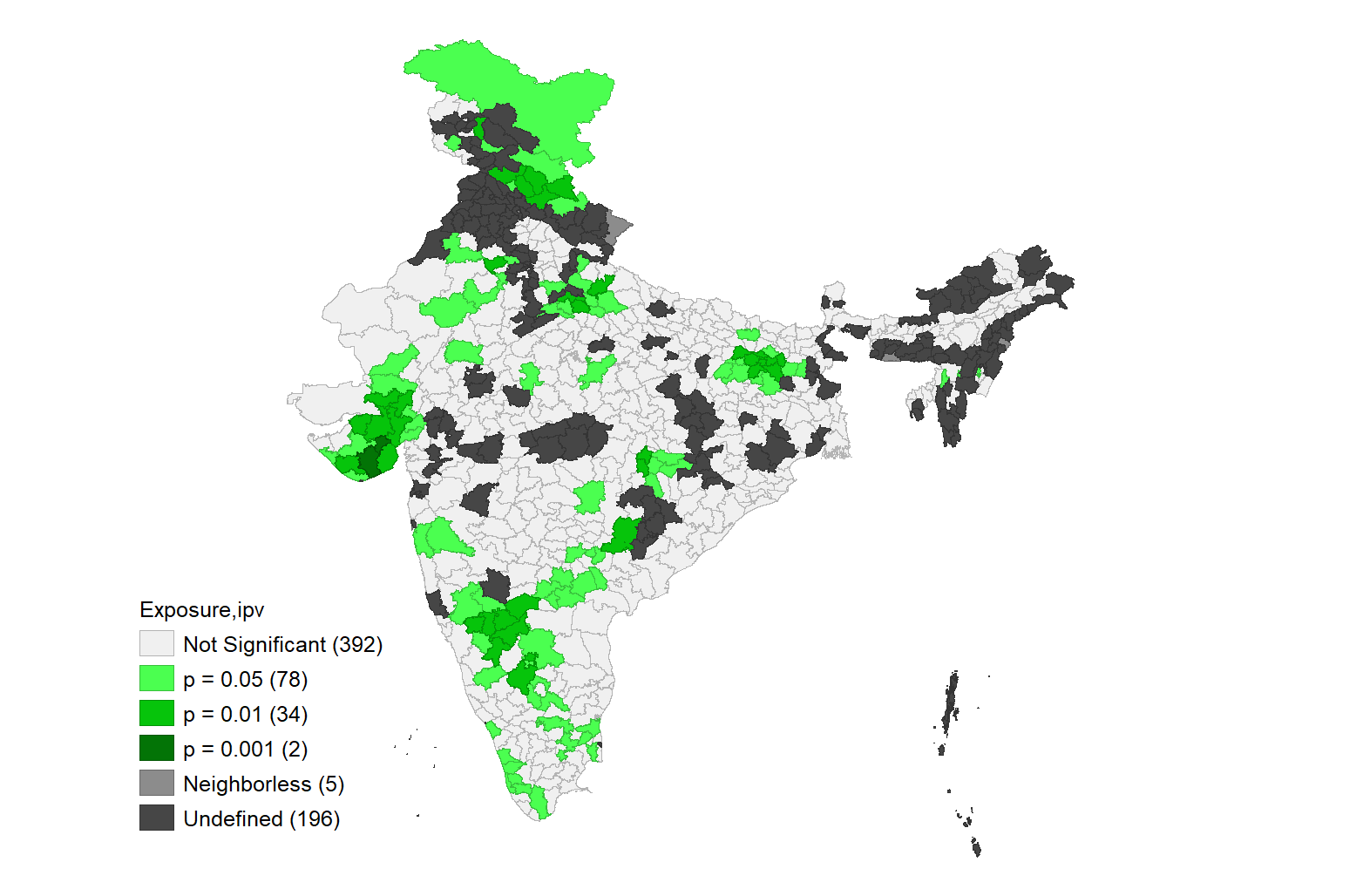


*Note: p-value for Global Moran’s I is 0.064*

Figure S.3. LISA Scatter Plot matrix and Significance Map for Miscarriage/Stillbirth


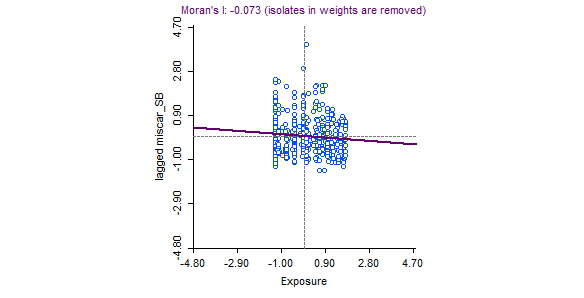

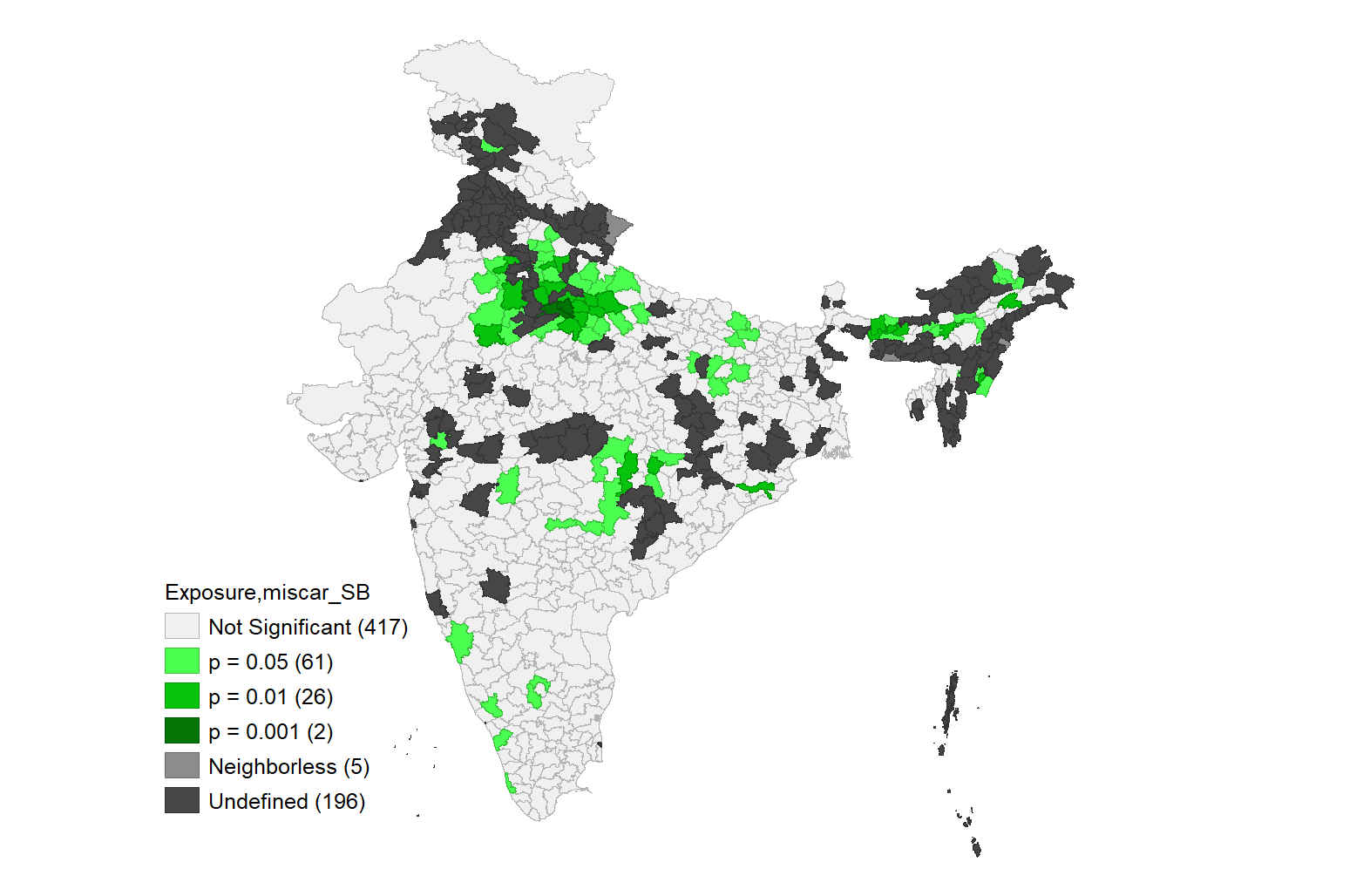


*Note: p-value for Global Moran’s I is 0.001*

Figure S.4. LISA Scatter Plot matrix and Significance Map for ANC Discontinuation


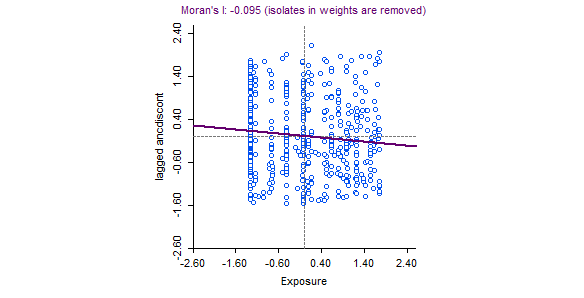

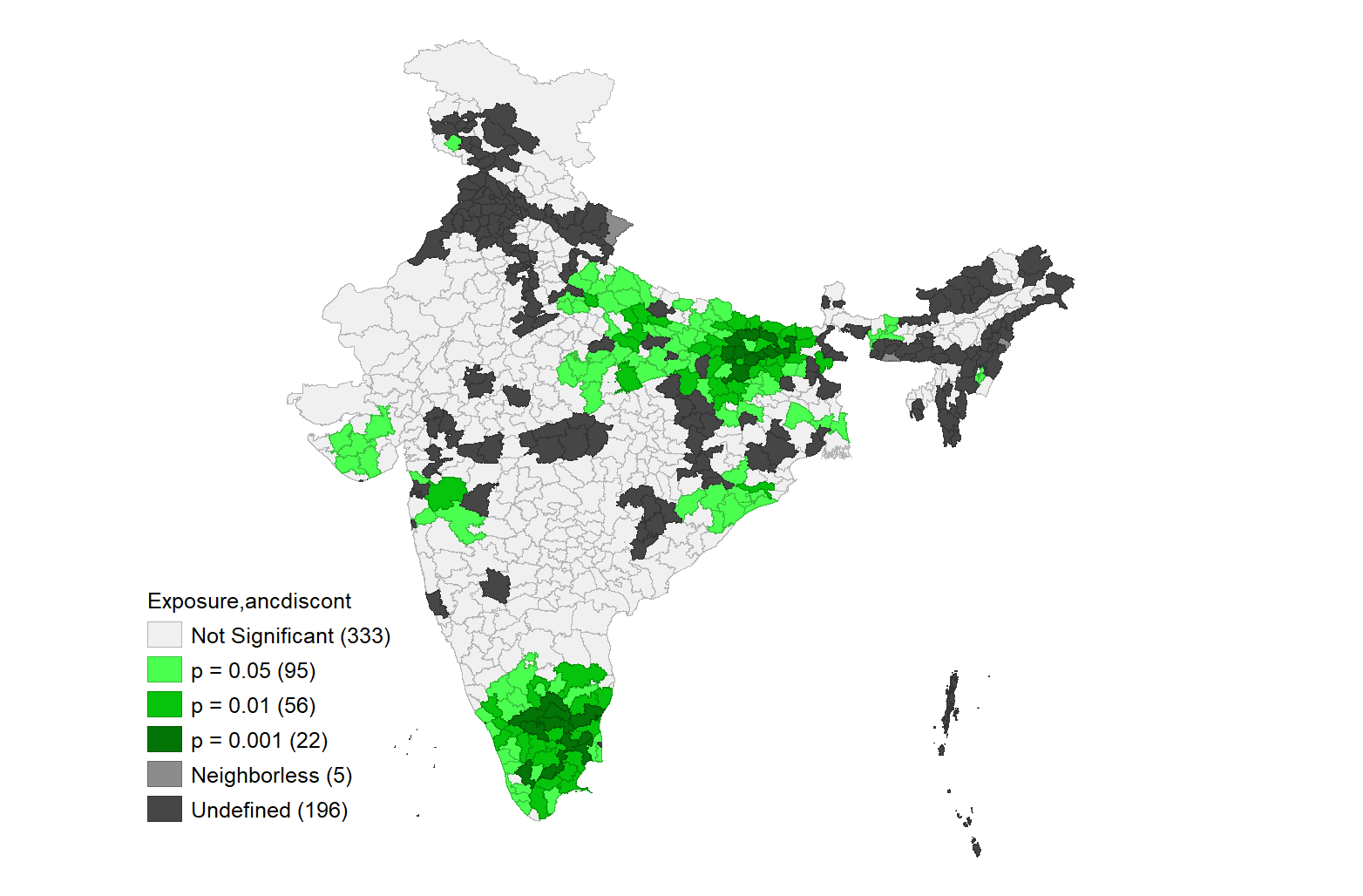


*Note: p-value for Global Moran’s I is 0.001*
